# Utilization of PET-CT in target volume delineation for three-dimensional conformal radiotherapy in patients with non-small cell lung cancer and atelectasis

**DOI:** 10.1186/2049-6958-8-21

**Published:** 2013-03-18

**Authors:** Li-Jie Yin, Xiao-Bin Yu, Yan-Gang Ren, Guang-Hai Gu, Tian-Gui Ding, Zhi Lu

**Affiliations:** 1 Department of Radiotherapy, Dalian Central Hospital, Dalian 116033, China; 2 China Petroleum Central Hospital, Langfang, 065000, China; 3 China Medical University, Shenyang, 110001, China

**Keywords:** Atelectasis, PET-CT, Non-small cell lung cancer, Target volume, Three-dimensional conformal radiotherapy

## Abstract

**Background:**

To investigate the utilization of PET-CT in target volume delineation for three-dimensional conformal radiotherapy in patients with non-small cell lung cancer (NSCLC) and atelectasis.

**Methods:**

Thirty NSCLC patients who underwent radical radiotherapy from August 2010 to March 2012 were included in this study. All patients were pathologically confirmed to have atelectasis by imaging examination. PET-CT scanning was performed in these patients. According to the PET-CT scan results, the gross tumor volume (GTV) and organs at risk (OARs, including the lungs, heart, esophagus and spinal cord) were delineated separately both on CT and PET-CT images. The clinical target volume (CTV) was defined as the GTV plus a margin of 6-8 mm, and the planning target volume (PTV) as the GTV plus a margin of 10-15mm. An experienced physician was responsible for designing treatment plans Plan_CT_ and Plan_PET-CT_ on CT image sets. 95% of the PTV was encompassed by the 90% isodose curve, and the two treatment plans kept the same beam direction, beam number, gantry angle, and position of the multi-leaf collimator as much as possible. The GTV was compared using a target delineation system, and doses distributions to OARs were compared on the basis of dose-volume histogram (DVH) parameters.

**Results:**

The GTV_CT_ and GTV_PET-CT_ had varying degrees of change in all 30 patients, and the changes in the GTV_CT_ and GTV_PET-CT_ exceeded 25% in 12 (40%) patients. The GTV_PET-CT_ decreased in varying degrees compared to the GTV_CT_ in 22 patients. Their median GTV_PET-CT_ and median GTV_PET-CT_ were 111.4 cm^3^ (range, 37.8 cm^3^-188.7 cm^3^) and 155.1 cm^3^ (range, 76.2 cm^3^-301.0 cm^3^), respectively, and the former was 43.7 cm^3^ (28.2%) less than the latter. The GTV_PET-CT_ increased in varying degrees compared to the GTV_CT_ in 8 patients. Their median GTV_PET-CT_ and median GTV_PET-CT_ were 144.7 cm^3^ (range, 125.4 cm^3^-178.7 cm^3^) and 125.8 cm^3^ (range, 105.6 cm^3^-153.5 cm^3^), respectively, and the former was 18.9 cm^3^ (15.0%) greater than the latter. Compared to Plan_CT_ parameters, Plan_PET-CT_ parameters showed varying degrees of changes. The changes in lung V_20_, V_30_, esophageal V_50_ and V_55_ were statistically significant (*Ps*< 0.05 for all), while the differences in mean lung dose, lung V_5_, V_10_, V_15_, heart V_30_, mean esophageal dose, esophagus Dmax, and spinal cord Dmax were not significant (*Ps*> 0.05 for all).

**Conclusions:**

PET-CT allows a better distinction between the collapsed lung tissue and tumor tissue, improving the accuracy of radiotherapy target delineation, and reducing radiation damage to the surrounding OARs in NSCLC patients with atelectasis.

## Background

Radiation is one of the important means for the treatment of non-small cell lung cancer NSCLC. In the process of radiotherapy, target area sketch is very important, and it has important significance in the treatment of patients with curative effect and prognosis. Most of the radiation treatment planning system is based on Computed Tomography (CT) image as a target area sketching and dose calculation basis. However, when NSCLC patients have atelectasis or obstructive pneumonia, it is difficult to distinguish the boundaries between incompletely expanded lung tissue and tumor tissue by conventional CT, which often results in inaccurate target delineation. As a consequence, insufficient dose coverage of the target volume or too much damage to normal tissue is caused. The advent of Positron emission tomography-Computed Tomography (PET-CT) can help overcome this problem. PET-CT is a fusion of functional information got by PET and anatomic information got by CT [1-3]. PET-CT can effectively identify the boundary between atelectasis region and lung cancer, make radiation target area precision, avoid unnecessary radiation injury and reduce radiation complications, thereby improving the radiation effect. The studies of Balogh *et al*. [4]. have shown that PET-CT imaging avoided the CT miscarriage of justice for tumor obstructive pneumonia or atelectasis organization, so that the gross tumor volume (GTV) narrowed, and found the transfer of lymph nodes which were not founded on CT, so that the GTV increased. Hoseok *et al*. [5] and Wang *et al*.[6] research results show that based on the PET - CT radiation plan can improve the outline of the GTV, helps to reduce the high dose lung and esophageal illuminated doses, may reduce the lung and esophageal radiation related to toxic reaction and improve the patient's quality of life, and in the same toxic reaction of cases is expected to further improve the target dose, improving the local control rate. The research indicates that the PET-CT improves the accuracy of the clinical stage, provides the basis for patients to choose the correct treatment approach, avoids the treatment select error induced by the mistakes of stage. At the same time, PET-CT can make target area sketch more accurate, better protect surrounding normal tissues, enhance the curative effect and reduce the radiation complications, thereby improving the patient's quality of life (Tables 1, 2 and 3).

In this study, we retrospectively analyzed the PET-CT imaging data for 30 NSCLC patients with atelectasis who underwent three-dimensional conformal radiotherapy and investigated the utilization of PET-CT in target volume delineation in these patients.

**Table 1:** Clinical characteristics of patients

**Clinical characteristic**	**Cases**
Age	
≥60 years	20
<60 years	10
Gender	
Male	19
Female	11
Histological type	
Squamous cell carcinoma	20
Adenocarcinoma	8
Adenosquamous carcinoma	2
Tumor location	
Central	21
Peripheral	9
TNM stage	
IIIA	14
IIIB	16

**Table 2:** GTV obtained from CT and PET-CT images in 30 patients

GTV (cm^3^)	**1**	**2**	**3**	**4**	**5**	**6**	**7**	**8**	**9**	**10**	**11**	**12**	**13**	**14**	**15**	**16**	**17**	**18**	**19**	**20**	**21**	**22**	**23**	**24**	**25**	**26**	**27**	**28**	**29**	**30**
CT	301.0	76.2	134.6	131.6	102.1	146.3	98.2	178.3	153.5	165.2	167.8	214.6	156.7	108.5	101.2	245.7	108.9	257.4	192.4	101.6	106.8	163.7	144.7	114.8	105.6	131.3	106.5	153.5	121.9	128.4
PET-CT	105.8	37.8	95.4	122.7	72.7	107.2	71.6	125.6	128.7	100.7	135.7	100.4	146.2	101.4	93.5	168.1	99.4	109.1	188.7	96.5	100.4	142.9	149.9	125.4	136.4	148.2	131.4	178.7	134.1	153.8

*GTV *Gross Tumor Volume, *CT *Computed Tomography, *PET-CT* Positron Emission Tomography-Computed Tomography.

**Table 3:** **Dose-volume histogram parameters for Plan**_**CT**_**and Plan**_**PET-CT**_

**Parameter**	**Plan**_**PET-CT**_	**Plan**_**CT**_	***t***	***P***
Lungs				
V_5 _(%)	46.2 ± 22.3	46.4 ± 22.5	-0.866	0.394
V_10 _(%)	37.6 ± 19.1	37.9 ± 19.6	-1.738	0.089
V_15 _(%)	26.7 ± 11.9	27.3 ± 15.4	-0.938	0.352
V_20 _(%)	18.1 ± 9.6	19.6 ± 10.2	-2.108	0.040
V_30 _(%)	13.2 ± 7.4	14.8 ± 8.5	-2.082	0.042
MLD (Gy)	13.1 ± 4.9	13.9 ± 5.3	-1.918	0.062
Heart				
V_30 _(%)	20.5 ± 17.4	21.6 ± 19.2	-0.676	0.502
MHD (Gy)	15.8 ± 9.1	16.7 ± 10.3	-0.659	0.512
Esophagus				
V_50 _(%)	19.6 ± 15.1	24.5 ± 18.4	-2.326	0.029
V_55 _(%)	15.4 ± 13.8	20.7 ± 17.6	-2.366	0.022
Dmax (Gy)	61.2 ± 10.2	61.7 ± 12.1	-0.083	0.936
Spinal cord				
Dmax (Gy)	40.3 ± 10.9	43.2 ± 11.5	-1.892	0.065

*Dmax *maximum dose, *MHD *Mean Heart Dose, *MLD *Mean Lung Dose, *V*_*x *_the percentage volume of organ receiving X Gy of dose, *MLD *Mean Lung Dose, *MHD *Mean Heart Dose, *Dmax *maximum dose.

## Methods

### Clinical data

Thirty NSCLC patients who underwent PET-CT at the First Affiliated Hospital of Dalian Medical University from August 2010 to March 2012 were included in this study. All patients were pathologically confirmed to have atelectasis. There were 19 males and 11 females. They ranged in age from 54 to 87 years, with a median age of 71 years. Of all patients, 20 had squamous cell carcinoma, 8 had adenocarcinoma, and 2 had adenosquamous carcinoma; 14 had TNM stage IIIA disease and 16 had stage IIIB disease. Inclusion criteria were: NSCLC patients who were willing to undergo radical three-dimensional conformal radiotherapy; those with lesions detectable by CT or other imaging modalities; histologically or cytologically confirmed NSCLC; standardized uptake value (SUV) ≥ 2.5; KPS score ≥ 70 points; and no pericardial effusion.

### Equipment and reagents

The Biograph 64 PET/CT system (number of slices = 64; number of detector rings = 39; Siemens, USA) equipped with the RDS Eclipse cyclotron was used in this study. ^18^F-fluorodeoxyglucose (FDG) with a radiochemical purity of > 96% was used.

### PET-CT procedure

All patients were asked to fast for 6 h. Prior to the PET-CT procedure, blood glucose levels were determined. ^18^F-FDG was then intravenously injected at a dose of 0.15 mCi/kg in patients whose blood glucose levels were in the normal range. Examinations started 60 min after injection, which consisted of a spiral CT scan followed by a PET scan during quiet breathing. CT data were used for attenuation correction of PET images. Images were then reconstructed using iterative methods, followed by multi-slice and multi-frame imaging. PET image reconstruction parameters were: slice thickness for PET image reconstruction = 5 mm, matrix = 168 × 168, slice thickness for CT image reconstruction = 5 mm, and cross-sectional resolution = 512 × 512. Finally, PET and CT data were transferred to the ADAC Pinnacle3TPS workstation where the data sets were fused automatically.

### Target volume delineation

The fused data sets were transferred to the Elekta TOMCON workstation for delineation of target volumes.

(1) Gross tumor volume (GTV): The GTV refers to clinically or radiologically demonstrable extent and location of tumors, including primary pulmonary lesions and metastatic lymph nodes. Lymph nodes with a short-axis diameter ≥ 1 cm were considered metastatic lymph nodes. Contours of primary pulmonary lesions and metastatic lymph nodes were first defined based on CT images on lung window (window width = 1600 Hu, window level = -600 Hu) and mediastinal window (window width = 400 Hu, window level = 20 Hu) settings to obtain GTV_CT_, then based on PET-CT fusion images (also including primary lesions and positive lymph nodes) to obtain GTV_PET-CT_. The threshold of 42% of maximum SUV (SUVmax) was used for tumor delineation. GTV_CT_ and GTV_PET-CT_ values were calculated automatically on the Elekta TOMCON workstation.

(2) Clinical target volume (CTV): The CTV includes the GTV and the range of subclinical lesions. In this study, a uniform margin of 8 mm was added around the GTV in adenocarcinoma cases, 6 mm in squamous cell carcinomas, and 6-8 mm in adenosquamous carcinoma cases depending on specific situations to form the CTV.

(3) Planning target volume (PTV): The PTV takes into consideration uncertainties caused by physiological displacement of organs, patient movements, and set-up errors that occur during each daily delivery of radiation. Internal target volume (ITV) was used to assess physiological displacement of organs, including the PTV and a margin compensating for daily positioning errors and internal motion of organs. The ideal CTV should result in the highest probability of CTV coverage, thus ensuring exposure of the CTV to maximum prescribed dose irradiation in each fraction of radiation. In this study, the PTV was defined by adding a margin of 10-15mm to the GTV.

### Delineation of organs at risk (OARs)

(1) Lungs: automatically delineated on the Elekta TOMCON workstation and then manually modified to exclude the trachea and bronchi.

(2) Heart: delineated from the bottom of the aortic arch to the bottom of the heart.

(3) Esophagus: delineated from the level of the cricoid cartilage to the area above the esophagogastric junction.

(4) Spinal cord: delineated slice by slice after adjusting CT window width and level to clearly demonstrate the spinal cord.

### Radiation treatment planning

Treatment planning was performed using the Elekta Precise Plan treatment planning system.

The PTV_CT_ and PTV_PET-CT_ were defined by adding a margin of 1.0-1.5 cm to the GTV_CT_ and GTV_PET-CT_. An experienced physician was responsible for designing three-dimensional conformal radiotherapy treatment plans Plan_CT_ and Plan_PET-CT_ on CT image sets. 95% of the PTV should be encompassed by the 90% isodose curve, and the two treatment plans should keep the same beam direction, beam number, gantry angle, and position of the multi-leaf collimator as much as possible. The prescribed dose was 2 Gy per daily fraction, 5 days per week, to a total dose of 60 Gy in 30 fractions. Dose constraints to OARs were lung V_20_ < 35%, heart D_1/3_ < 50 Gy, spinal cord Dmax< 45Gy, and esophagus Dmax< 60Gy.

### Main indicators and parameters

(1) GTV_CT_ and GTV_PET-CT_ calculated automatically on the workstation;

(2) Dose-volume histogram (DVH) parameters, including mean lung dose (MLD), lung V_5_, V_10_, V_15_, V_20_ and V_30_, mean heart dose (MHD), heart V_30_, esophageal V_50_ and V_55_, esophageal Dmax, and spinal cord Dmax.

### Statistical analysis

Statistical analysis was performed using SPSS 19.0 software. Comparisons between two groups were performed using the *t*-test. Data were expressed as mean ± standard deviation (SD). *P* <0.05 was considered statistically significant.

## Results

### GTV

The GTV_CT_ and GTV_PET-CT_ had varying degrees of change in all 30 patients, and the changes in GTV_CT_ and GTV_PET-CT_ exceeded 25% in 12 (40%) patients.

The GTV_PET-CT_ decreased in varying degrees compared to the GTV_CT_ in 22 patients. Their median GTV_PET-CT_ and median GTV_PET-CT_ were 111.4 cm^3^ (range, 37.8 cm^3^-188.7 cm^3^) and 155.1 cm^3^ (range, 76.2 cm^3^-301.0 cm^3^), respectively, and the former was 43.7 cm^3^ (28.2%) less than the latter.

The GTV_PET-CT_ increased in varying degrees compared to the GTV_CT_ in 8 patients. Their median GTV_PET-CT_ and median GTV_PET-CT_ were 144.7 cm^3^ (range, 125.4 cm^3^-178.7 cm^3^) and 125.8 cm^3^ (range, 105.6 cm^3^-153.5 cm^3^), respectively, and the former was 18.9 cm^3^ (15.0%) greater than the latter.

**Figure 1:**
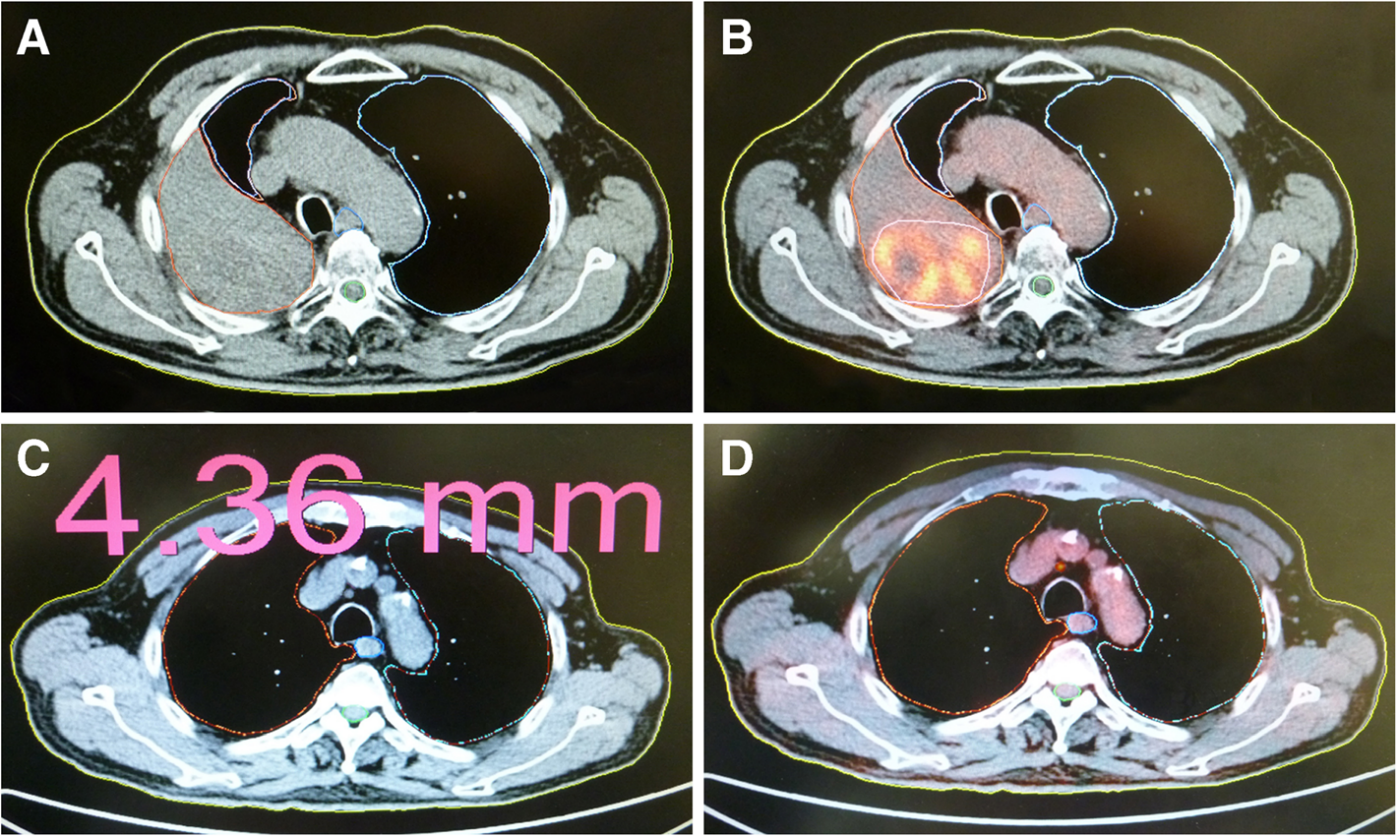
**Comparison between conventional CT and PET-CT image. A**: By conventional CT, it was difficult to distinguish the boundaries between incompletely expanded lung tissue and tumor tissue with consequent excessive target delineation. **B: **By PET-CT, the areas of high metabolic activity indicated the presence of tumors. The target volume was obviously smaller than that on conventional CT image. **C: **On conventional CT image, the mediastinal lymph node had a short-axis diameter of 4.36 mm and was not considered a metastatic lymph node. **D: **On PET-CT image, the mediastinal lymph node showed high metabolic activity and was considered a metastatic lymph node.

The main reason for the decrease in the GTV_PET-CT_ relative to the GTV_CT_ is that PET-CT allowed distinguishing tumor tissue from collapsed lung tissue based on their difference in functional metabolism and reducing the target volumes (Figure 1A and B). In contrast, it was difficult to distinguish the boundaries between incompletely expanded lung tissue and tumor tissue by conventional CT, which resulted in excessive target delineation.

The main reason for the increase in the GTV_PET-CT_ relative to the GTV_CT_ is that PET-CT allowed detecting metastatic lymph nodes that could not be identified by conventional CT and increasing the target volumes (Figure 1C and D).

### OARs

Compared to Plan_CT_ parameters, Plan_PET-CT_ parameters showed varying degrees of change. The changes in lung V_20_, V_30_, esophageal V_50_ and V_55_ were statistically significant (*Ps*< 0.05 for all), while the differences in MLD, lung V_5_, V_10_, V_15_, heart V_30_, MHD, esophageal Dmax, and spinal cord Dmax were not statistically significant (*Ps*> 0.05 for all).

## Discussion

Generally, three-dimensional conformal radiotherapy treatment planning is based on CT images; however, the information provided by CT data often cannot meet the requirements of target volume delineation [7]. In recent years, PET-CT has been increasingly used in clinical practice to delineate the target volumes for radiotherapy of lung cancer. PET-CT has an accuracy superior to that of conventional CT and other imaging modalities. Deniaud-Alexandre *et al*. [8] delineated the GTV in 92 NSCLC patients by PET-CT and found that the GTV_PET-CT_ was reduced in 23% of the patients and increased in 26% of cases compared to GTV_CT_, and 21 patients had a GTV change of ≥ 25%. In this study, we found that all 30 patients had varying degrees of changes in the GTV_PET-CT_ and GTV_CT_, including 12 (40%) patients who had a change over 25%. This result is consistent with those reported by Deniaud-Alexandre *et al*. and Ceresoli *et al*. [9].

Although it is important to meet the requirements of target dose distribution, serious complications of radiation therapy caused by too large irradiated volume or too high dose to OARs should also be avoided. A given radiation treatment plan in which the target irradiation volume and the PTV fit well and the dose is evenly distributed is still unacceptable when the dose to OARs exceeds the tolerable dose of the organ or the irradiated volume of OARs is too large, because the implementation of this treatment plan will cause great damage to normal tissue and serious radiotherapy complications. Bradley *et al*. [10] contoured the GTV from the CT and PET-CT data sets in 26 NSCLC patients and found that, in three patients with atelectasis, the GTV and PTV obtained from PET-CT images were significantly reduced compared to those obtained from CT images, the MLD decreased from 14.83 Gy to 12.93 Gy, and lung V_20_ decreased from 25.33% to 21.33%. They also discovered that the MLD and mean esophageal dose increased with the increase in the GTV in 11 patients whose target volumes increased as a result of additional detection of metastatic lymph nodes. Van Der Wel *et al*. [11] contoured the target volumes by PET-CT and found that the GTV of the lymph nodes, lung V_20_, MLD, esophageal V_45_ and V_55_ decreased. At the same level of radiation toxicity, radiation dose and tumor control rate were improved. As a result, the efficacy of radiation therapy was enhanced. In the present study, we found that Plan_PET-CT_ parameters showed varying degrees of change compared to Plan_CT_ parameters. The changes in lung V_20_, V_30_, esophageal V_50_ and V_55_ were statistically significant (*Ps*< 0.05 for all), while the differences in MLD, lung V_5_, V_10_, V_15_, heart V_30_, MHD, esophageal Dmax, and spinal cord Dmax were not significant (*Ps*> 0.05 for all).

Acute radiation-induced lung injury is a kind of lymphocytic alveolar inflammation caused by direct radiation damage and body’s immune response. The severity of lung functional injury after radiotherapy is closely related to the irradiated volume. The dose-volume histogram (DVH) offers a range of physical parameters for the evaluation of radiotherapy-induced lung injury in lung cancer patients after three-dimensional conformal radiotherapy. The V_20_ is currently the most widely used parameter for clinical evaluation of treatment plans. However, the results obtained on factors associated with acute radiation-induced lung injury are different among different studies. In a study involving 99 NSCLC patients performed by Graham *et al*. [12], univariate analysis showed that the V_20_ and MLD were closely associated with the development of acute radiation-induced lung injury (grade 2 or higher), and multivariate analysis showed that the V_20_ was the only independent predictive factor for acute radiation-induced lung injury. This result is consistent with that obtained by Tsujino *et al*. [13]. In a study conducted by Zhang *et al*. [14], univariate analysis indicated that the MLD, mean dose to the affected lung, and V_20_ were factors associated with the development of acute radiation-induced lung injury, and multivariate analysis indicated that only the mean dose to the affected lung is the independent risk factor. Studies performed by Hernando *et al*. [15], Claude *et al*. [16], and Kim *et al*. [17] demonstrated that the V_30_ was a factor associated with the development of acute radiation-induced lung injury. A recent study by Wang *et al*. [18] showed that the V_5_ is also associated with the development of acute radiation-induced lung injury, suggesting that V_5_ as a dose-volume constraint should be fully taken into account in designing radiation treatment plans. In our study, all Plan_PET-CT_ parameters had varying degrees of decrease compared to Plan_CT_ parameters, indicating that delineation of the target volumes for radiotherapy by PET-CT can help reduce the incidence of acute radiation-induced lung injury in NSCLC patients.

Acute radiation-induced esophageal injury usually occurs about two weeks after the start of radiotherapy. In recent years, there have been more and more studies investigating factors associated with the development of esophageal injury in patients undergoing three-dimensional conformal radiotherapy for NSCLC, although the parameters used and the conclusions drawn varied among different studies. Kim *et al*. [19] suggested that the V_60_ was an important parameter to predict acute radiation esophagitis (grade 3 or higher). Algara *et al*. [20] found that the V_50_ was the most valuable predictor. Topkan *et al*. [21] indicated that the V_55_ was the only relevant dosimetric parameter. More studies indicated that the V_55_ was likely to be the most valuable parameter for predicting acute radiation-induced esophageal injury. Our results indicated that the decrease in esophageal V_55_ obtained using PlanPET-CT was statistically significant (p < 0.05) compared to that obtained using PlanCT, suggesting that delineation of the target volumes for radiotherapy by PET-CT can help reduce the incidence of acute radiation-induced esophageal injury in NSCLC patients.

Radiation myelitis is a myelopathy that develops following spinal cord exposure to therapeutic radiation [22]. Due to the combined effects of a variety of factors, neuronal degeneration and necrosis occur. The development of radiation myelitis is associated with exposure of normal spinal cord tissue to high-dose radiation [23,24]. In our study, spinal cord Dmax obtained using PlanPET-CT decreased compared to that obtained using PlanCT, but the difference was not statistically significant. More emphasis should be put on the prevention of radioactive myelitis, and radiation dose to the spinal cord must be strictly controlled during radiotherapy.

Radiation damage to the heart is mainly manifested as ECG abnormalities, especially ischemic ST-T changes. The incidence of heart injury will significantly increase if 1/3 of the heart volume receives 70 Gy, 2/3 of the heart volume receives 55 Gy, or the whole heart receives 50 Gy. Our study suggests that PET-CT can help protect from cardiac injury to a certain extent, although the difference was not obvious between the two groups.

## Conclusions

In summary, the results of this study show that PET-CT fusion image is beneficial to target area sketch in patients merged with atelectasis of non-small cell lung cancer, and optimization of precise radiotherapy planning. By lowering the irradiation dose of the surrounding normal lung tissue and avoiding missing the irradiation of target area, the utilization of PET-CT can reduce the probability of occurrence of acute radiation-induced lung injury and acute radiation-induced esophageal injury. Also, the utilization of PET-CT has a protective effect on the heart and spinal cord. It brings the benefits to the lung cancer patients. Therefore, we believe that PET-CT will play more and more important role in radiotherapy of NSCLC.

## Abbreviations

3D-CRT: Three-dimensional conformal radiotherapy; CTV: Clinical target volume; DHV: Dose volume histograms; GTV: Gross tumor volume; ITV: Internal target volume; PTV: Planning target volume; MHD: Mean heart dose; MLD: Mean lung dose; OAR: Organs at risk; SUV: Standardized uptake value.

## Competing interests

The authors declare that they have no competing interests.
